# Adenomyoepithelioma with Ductal Carcinoma In Situ: A Case Report and Review of the Literature

**DOI:** 10.1155/2013/521417

**Published:** 2013-02-25

**Authors:** Sanjay Warrier, Sang Hwang, Martha Ghaly, Alex Matthews

**Affiliations:** ^1^Department of Surgery, The Prince of Wales Hospital, Barker Street, Randwick, Sydney, NSW 2031, Australia; ^2^The University of New South Wales, Sydney, NSW 2052, Australia

## Abstract

Adenomyoepithelioma (AME) with microglandular adenosis-like growth pattern and superimposed ductal carcinoma in situ (DCIS) was identified in a 55-year-old female after biopsy of an atypical lesion identified through routine breast screening. A literature review reveals that this association has rarely been described.

## 1. Introduction

Adenomyoepitheliomas (AMEs) are rare neoplastic lesions with biphasic proliferation and dedifferentiation of both glandular and myoepithelial cells [1, 2]. Although generally considered to be benign, malignant AMEs have been reported [3]. The association of AME with ductal carcinoma in situ (DCIS) has rarely been reported in the literature with only two previously reported cases [4, 5].

## 2. Case Report

A 55-year-old female presented to a breast screening program, BreastScreen, at Royal Hospital for Women, Sydney, Australia. The patient noted a periareolar lump in her left breast and had a maternal aunt who was diagnosed with ductal carcinoma of the breast at 60 years of age. On examination, there was a palpable periareolar irregularity in the 3 o'clock position. No other clinical abnormalities were noted. Screening mammography (MMG) did not detect any abnormal lesion.

Ultrasound (US) examination confirmed a 2 cm left breast lesion at the 3 o'clock position, 1 cm from the areola. It showed a solid mass with acoustic shadowing and a dilated duct. Fine needle aspiration biopsy was inconclusive demonstrating “*benign groups of cohesive and atypical ductal cells with associated myoepithelial cells*.*”*

Consequently, a hookwire-guided excisional biopsy was performed. Microscopy showed

*…a highly unusual glandular lesion with an infiltrative architecture resembling microglandular adenosis. The lesions consist of round glands and solid clusters consisting both epithelial and myoepithelial cells… In addition, there are areas of epithelial atypia, mitotic activity and punctate necrosis resembling intermediate grade DCIS.*

**Figure 1:**
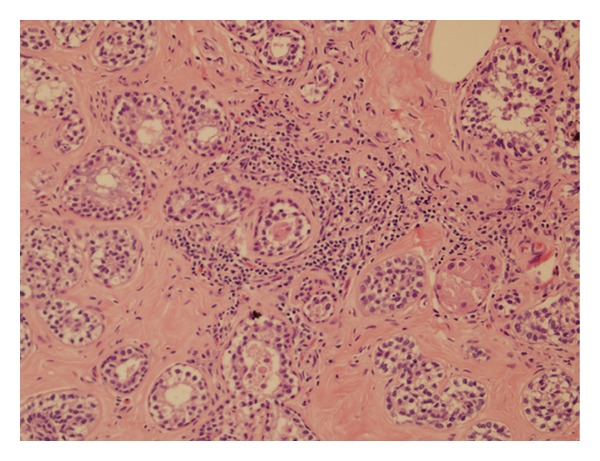
Hookwire localised excision biopsy showed both epithelial and myoepithelial cells on haematoxylin and eosin staining (H&E ×200).

**Figure 2:**
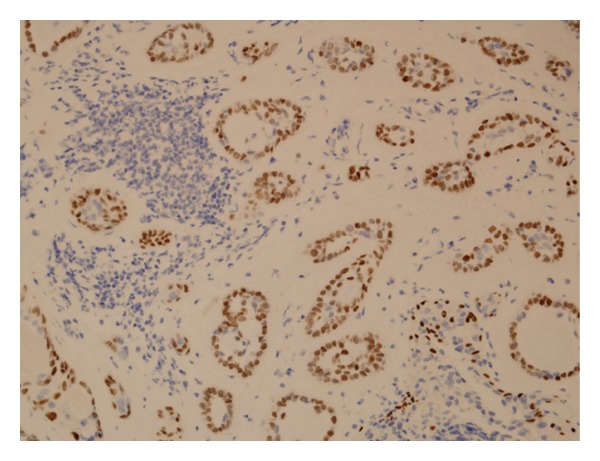
Immunohistochemistry for p63 was positive, demonstrating the presence of myoepithelial cells (×200).

This patient's histopathology demonstrating AME is shown in Figures 1 and 2.

Discussion at a multidisciplinary meeting provided consensus to perform a wide local excision (WLE) and sentinel lymph node biopsy (SNLB). The pathology on WLE revealed multinodular AME with high grade DCIS extending to the lateral margin. The three sentinel lymph nodes biopsied were negative. 

On the basis of positive margins for DCIS, mastectomy with immediate reconstruction or breast-conserving surgery with adjuvant radiotherapy was offered to the patient. The patient opted for mastectomy with immediate reconstruction, and this was performed without any complications. Final pathology revealed completely excised AME with DCIS. 

## 3. Discussion

AME arises from myoepithelial and epithelial cells in the normal breast lobules and ducts [6]. These tumours are characterised by biphasic proliferation of epithelial and myoepithelial cells. These lesions were first described by Hamperl [7] in 1970 and further classified by Tavassoli [3] in 1991 into tubular, papillary, and solid subtypes. 

The biphasic structure of AME is formed by cuboidal to columnar epithelial lined tubules, which is surrounded by a layer of myoepithelial cells with prominent cytoplasm [8]. Seifert et al. [9] concluded that AME may be histologically identical to epithelial myoepithelial carcinoma (EMEC) of the salivary glands and postulated that the mostly benign behaviour of AME was related to its smaller average size. 

AME generally runs a benign course but malignant AME can metastasise [10]. Metastases have been described in sites including bone (ribs, spine, and jaw), lung, brain, and regional lymph nodes [11, 12]. Malignancy can develop within the epithelial component, the myoepithelial component, or in both components [12]. 

In this case, the malignancy arising in the epithelial component of AME was a high grade DCIS, and this association is extremely rare.  Table 1 illustrates the two prior published cases of AME with DCIS. The DCIS can arise as a result of the malignant transformation of the epithelial component of AME [4] or as a separate, distinct primary tumour. The presence of DCIS can further guide surgical and adjuvant treatment.

Surgical options vary depending on the size, location, focality, and associated pathology. Wide local excision with clear margins is generally recommended but the degree of margins required is unknown as some cases of AME show rapid local recurrences [13]. However, AMEs demonstrate a propensity for haematogenous rather than nodal spread, and it has previously been proposed that SNLB or axillary dissection can lead to overtreatment [14]. 

In summary, we report a rare association of AME with DCIS and MA-like growth pattern which was successfully treated with mastectomy with reconstruction and adjuvant radiotherapy. The patient is well with no local recurrence or metastasis at one-year followup. 

## Figures and Tables

**Table 1:** Summary of cases of adenomyoepithelioma with ductal carcinoma in situ of the breast.

Authors	Year	Age	Site	Size (cm)	Surgical treatment	Final pathology	Followup
Present case	2012	55	L	2	Mastectomy	AME + DCIS	1 year, disease-free
Han and Peng [4]	2010	55	L	3.5	Mastectomy	AME + DCIS	3.5 years, disease-free
Ng [5]	2002	41	R	1.3	WLE	AME + DCIS	n/a

WLE: wide local excision, AME: adenomyoepithelioma, and DCIS: ductal carcinoma in situ.
